# An endophyte from salt-adapted Pokkali rice confers salt-tolerance to a salt-sensitive rice variety and targets a unique pattern of genes in its new host

**DOI:** 10.1038/s41598-020-59998-x

**Published:** 2020-02-24

**Authors:** Megha Hastantram Sampangi-Ramaiah, Jagadheesh, Prajjal Dey, Shridhar Jambagi, M. M. Vasantha Kumari, Ralf Oelmüller, Karaba N. Nataraja, Kundapura Venkataramana Ravishankar, G. Ravikanth, R. Uma Shaanker

**Affiliations:** 1 School of Ecology and Conservation, University of Agricultural Sciences, GKVK, Bangalore, 560065 India; 2 Friedrich-Schiller – University, Institute of General Botany and Plant Physiology, Dornbuger Str. 159, 07743 Jena, Germany; 3 Department of Crop Physiology, University of Agricultural Sciences, GKVK, Bangalore, 560065 India; 4 Division of Biotechnology, ICAR - Indian Institute of Horticultural Research, Hessaraghatta Lake Post, Bengaluru, 560089 India; 5 Ashoka Trust for Research in Ecology and the Environment, Royal Enclave, Srirampura, Jakkur Post, Bangalore, 560064 India

**Keywords:** Molecular biology, Plant sciences

## Abstract

Endophytes, both of bacterial and fungal origin, are ubiquitously present in all plants. While their origin and evolution are enigmatic, there is burgeoning literature on their role in promoting growth and stress responses in their hosts. We demonstrate that a salt-tolerant endophyte isolated from salt-adapted Pokkali rice, a *Fusarium sp*., colonizes the salt-sensitive rice variety IR-64, promotes its growth under salt stress and confers salinity stress tolerance to its host. Physiological parameters, such as assimilation rate and chlorophyll stability index were higher in the colonized plants. Comparative transcriptome analysis revealed 1348 up-regulated and 1078 down-regulated genes in plants colonized by the endophyte. Analysis of the regulated genes by MapMan and interaction network programs showed that they are involved in both abiotic and biotic stress tolerance, and code for proteins involved in signal perception (leucine-rich repeat proteins, receptor-like kinases) and transduction (Ca^2+^ and calmodulin-binding proteins), transcription factors, secondary metabolism and oxidative stress scavenging. For nine genes, the data were validated by qPCR analysis in both roots and shoots. Taken together, these results show that salt-adapted Pokkali rice varieties are powerful sources for the identification of novel endophytes, which can be used to confer salinity tolerance to agriculturally important, but salt-sensitive rice varieties.

## Introduction

Salinity stress is one of the most devastating abiotic stresses that affect growth, development and productivity of major crops. A soil is termed saline if its osmotic pressure is approximately 0.2 M Pa with an electrical conductivity of 4 dS/m or more (equivalent to ~40 mM NaCl)^1,2^. It is estimated that at least 50% of the arable land worldwide will be salt affected by the year 2050 and this is expected to further increase due to global climate change^3^. Rice *(Oryza sativa* L.), a major staple food crop, is one of the most salt-sensitive cereals^4^. In the Indo-Gangetic Basin in India, an estimated 45% loss in rice production was attributed to salinity stress alone^5,6^.

The responses of plants to salinity stress are often multi-faceted and complex. In the initial phase, salinity stress manifests itself as osmotic stress with reduction in water uptake by plants. This triggers a range of metabolic and molecular cascades such as inhibition of cell expansion, stomatal conductance, photosynthetic activity, abscisic acid (ABA)-mediated responses followed by stimulation of the SOD and peroxidase activities as well as accumulation of osmolytes like proline. The later phase of salinity stress leads to ionic stress, which is due to the alterations in the Na^+^/K^+^ and Na^+^/Ca^2+^ ratios because of the accumulation of both Na^+^ and Cl^−^. This promotes the production of reactive oxygen species (ROS) in cells and leads to oxidative stress^7^.

In recent years, an increasing number of studies have focused on the role of endophytes in alleviating salinity stress in plants. Endophytes constitute an important component of the plant microbiome and comprise of both bacteria and fungi. Ubiquitously present in all plants, they do not cause disease symptoms in their hosts but often promote their performance such as growth and resistance, in particular under abiotic and biotic stresses. The endophytes are often adapted to extreme habitats and might have developed strategies to activate stress tolerance responses in their host plants in which they reside^8,9^.

One of the most widely studied root endophyte, *Piriformospora indica*, has been reported to promote growth in a number of plant systems under abiotic stresses including salinity stress^10^. In *Arabidopsis*, *P. indica* maintains the Na^+^ and K^+^ homeostasis under salt stress^11^. Two bacterial endophytes, *Bacillus subtilis* and *Mesorhizobium ciceri*, confer salt tolerance to chickpea by decreasing H_2_O_2_ concentrations and increasing proline content^12^. *Pseudomonesa fluorescens* and *P. migulae* ameliorate salinity stress in tomato plants by increasing the 1-aminocyclopropane-1-carboxylate deaminase activity, the key enzyme for ethylene biosynthesis^13^. Asaf *et al*. (2018) demonstrated *Aspergillus flavus* CHS1-mediated salinity tolerance in *Glycine max*. L through the stimulation of the antioxidative system and endogenous hormone levels in the host^14^. In tomato, the endophyte, *Trichoderma harzianum* alters the expression level of 1243 genes in the roots^15^. Bajaj *et al*. (2018) showed that colonization of soybean plants by *P. indica* resulted in the stimulation of genes associated with the phenylpropanoid and lignin pathways, both of which are known to play an important role in oxidative stress tolerance^16^. However, the molecular mechanisms through which endophytes modulate these processes in their host are little understood^17^.

In this study, we examine the endophytic fungal assemblage of salt-adapted Pokkali land races and evaluate their ability to tolerate salinity stress. The Pokkali rice is a traditional salt-tolerant variety grown in parts of coastal Kerala, India, during the months of June to November. Because of the traditional practice, the variety has been conferred a Geographical Indicator (GI) tag^18^. One of the identified endophytic fungus, which was tolerant to high concentrations of salt, was transferred to the salt-sensitive rice variety IR-64. We demonstrate that the colonized IR-64 plants are not only morphometrically larger but also more tolerant to salt stress than the un-colonized control, and this is associated with an altered expression of a specific combination of genes. The results of these experiments are particularly novel and important because they not only open up exciting possibilities of using an endophytic route towards mitigating crop stress but also in understanding the underlying molecular basis of plant-endophyte interaction. The latter could inspire further studies into numerous plant-endophyte interactions reported in literature and seek if there is some commonalities to the way endophytes interact and lend habitat adapted symbiotic benefits to the plants.

## Results

### Isolation and characterization of fungal endophytes

Endophytes were isolated from 720 leaf and root segments as well as seeds from the salt-sensitive IR-64 variety and salt-tolerant Pokkali rice varieties. A total of 494 endophytic fungal isolates were obtained, 49.40% derived from the seeds, 29.95% from the roots and 20.65% from the shoots. The colonization frequency ranged from 46.6% to 96.6%, with the highest obtained in shoot segments of VTL-6 and the least in shoot tissue of VTL-4 (Supplementary file 1). Based on colony characteristics, the 494 isolates were categorized into 41 OTUs. Highest number of OTUs (11) was obtained for the variety IR-64 while the lowest number (5 OTUs) was obtained for VTL-6 (Supplementary file 1).

The homology search of the *ITS* region revealed that the majority of the OTUs belonged to *Gibberella intermedia* and *Fusarium solani* (12 OTUs and 7 OTUs, respectively) (Supplementary file 1). A few fungi, such as *Botryosphaeria dothidea* and *Alternaria alternata*, could only be isolated from salt-adapted Pokkali genotypes (Supplementary file 2).

### Evaluation of the fungal isolates for salinity stress tolerance

**Figure 1:**
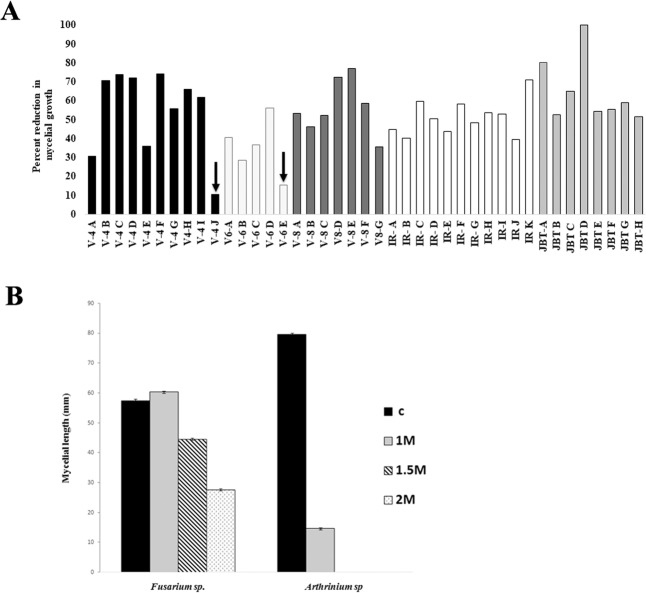
Mycelial growth: (**A**) Percent reduction in mycelial growth of 41 OTUs from salt adapted and sensitive genotypes subjected to 1 M NaCl. Arrow indicates highly salt tolerant OTUs. (V-4, V-6 and V-8 refer to isolates from Pokkali rice land races VTL-4, VTL-6 and VTL-8 respectively. IR refers to the isolates from IR-64 and JBT refers to isolate from JBT 36/14). (**B**) Mycelial growth of endophytic fungi, *Fusarium sp*., and *Arthrinium* sp. at different concentration of NaCl. Line over bars denote SD(±).

Growth of most of the endophytic fungi decreased with increase in NaCl concentration in the medium. V-4J (*Fusarium sp*.) isolated from VTL-4 showed only 10% reduction in mycelial growth on media with 1 M NaCl. On the other hand, growth of *Arthrinium* sp. hyphae isolated from JBT 36/14 was completely inhibited on 1 M NaCl (Fig. 1A). These two isolates were used for further investigations and referred to as salt-tolerant and salt-sensitive endophytes, respectively. When exposed to media with 1.5 M and 2 M NaCl, the salt-sensitive *Arthrinium* sp. endophyte failed to grow, while the salt-tolerant *Fusarium sp*. endophyte grew even on 1.5 M and 2 M NaCl media (diameter of fungal hyphae on plates: 57.3 ± 0.6 mm on media without NaCl; 44.7 ± 0.3 mm on 1.5 M NaCl; 27.7 ± 0.3 mm on 2.0 M) (Fig. 1B, Supplementary file 3).

### Fusarium sp. confers salinity tolerance to IR-64 seedlings

**Figure 2:**
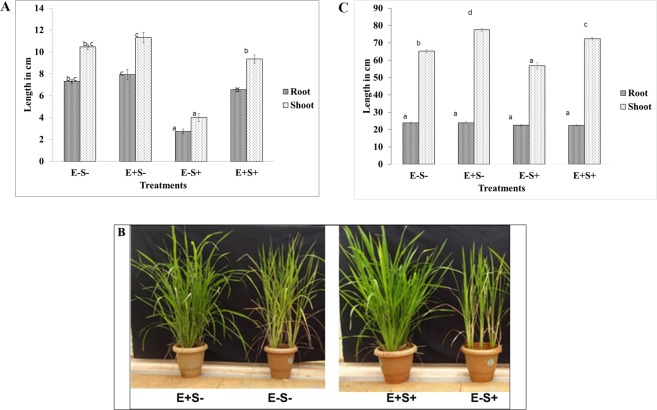
(**A**) Root and shoot growth of 10 day old IR-64 seedlings under different treatments (E−S−; E+S−; E−S+; E+S+ where E +/E−: Presence/absence of *Fusarium sp*. and S+/S−: Presence/absence of salinity stress (150 mM NaCl). (**B**) Phenotypic growth of 50 day old IR-64 plants as influenced by endophyte colonization under control (left) and under 4 ds/m stress (right). (**C**) Root and shoot growth of 50 day old IR-64 plants subjected to different treatments (E−S−; E+S−; E−S+; E+S+ where E+/E−: Presence/absence of *Fusarium sp*. and S+/S−: Presence/absence of salinity stress (4 ds/m). Line on bars indicates SEm (±). One-way ANOVA was done for root and shoot separately. Means with dissimilar letters are significant at P < 0.05 (Tukey).

Seeds of the salt-sensitive IR-64 variety were treated with the *Fusarium sp*. endophyte and the performance of the seedlings analyzed on agar plates with and without NaCl. The fungus induced a significant increase in root and shoot growth in 10-day-old seedlings under salinity stress, when compared to seedlings which were grown without the endophyte. The growth-promoting effect was not detectable for seedlings, which were grown without salt stress (Fig. 2A), although root colonization was detectable under both conditions (Supplementary file 3). When colonized and uncolonized IR-64 plants were grown in the greenhouse, the fungus induced a significant increase in shoot growth under both salinity stress and control conditions (Fig. 2B,C). However the fungus did not significantly alter the root growth under both salinity and control condition (Fig. 2C). The mean tiller number of plants under salt stress increased from 3.5/plant in plants not treated with the endophyte compared to 10.5/plant in those treated with the endophyte (p < 0.05; Table 1). There was however, no significant difference in the tiller number per plant due to endophyte treatment under control, non-stress conditions (Table 1). The effect of the endophyte colonization on the total biomass of 50-day old plant was examined. Colonization significantly enhanced the biomass both under control and salt stress conditions (p < 0.05; Table 1). These results clearly indicate that *Fusarium sp*. promotes the performance of IR-64 plants, in particular, under salinity stress.

**Table 1:** Growth, physiological and biochemical responses of IR-64 plants subjected to different treatments (E+/E−: Presence/absence of *Fusarium sp*.; S+/S−: Presence/absence of Salinity stress) (^a^50 day old plants; ^b^10 day old seedlings). One-way ANOVA was done separately for E−S- and E+S− and for E−S+ and E+S+. Results are the mean of three replication ± SEm. Asteriks indicate significant difference between means within each of the above pairs of comparison at P < 0.05.

Treatments/Parameters	E−S−	E+S−	E−S+	E+S+
**Growth parameters**				
Tiller number/plant^a^	6.83 ± 0.490	11.17 ± 1.705	3.5 ± 0.235	10.5 ± 0.848*
Total biomass (g/plant)^a^	19.7 ± 0.567	25.5 ± 1.479*	11.6 ± 0.139	15.6 ± 0.597*
**Physiological parameters**				
Total chlorophyll content (mg g^−1^ fw)^a^	0.05 ± 0.007	0.11 ± 0.007*	0.01 ± 0.001	0.04 ± 0.007*
Assimilation rate (μ mol m^−2^ s^−1^)^a^	12.97 ± 0.808	15.67 ± 0.533	8.80 ± 1.031	13.97 ± 0.998*
Stomatal conductance (m mol m^−2^ s^−1^)^a^	0.27 ± 0.046	0.25 ± 0.046	0.11 ± 0.026	0.16 ± 0.031
Transpiration rate (mol m^−2^ s^−1^)^a^	3.84 ± 0.410	3.15v0.010	2.11 ± 0.332	2.69 ± 0.363
**Biochemical parameters**				
SOD activity^b^	50.7 ± 1.142	46.57 ± 7.730	43.88 ± 5.999	25.69 ± 7.627
MDA content (µ mol g^−1^)^b^	7.49 ± 0.049	9.28 ± 0.049*	11.07 ± 0.049	12.11 ± 0.049*
Proline content (µ mol g^−1^)^b^	0.17 ± 0.024	0.07 ± 0.002*	0.60 ± 0.000	0.31 ± 0.000*

The effect of the fungus on the performance of the salt-exposed plants was further analyzed for physiological parameters describing the fitness of the plants. The endophyte-treated plants had significantly higher total chlorophyll content under both control and salt stress conditions (p < 0.05; Table 1). On the other hand, the net carbon assimilation rates were significantly higher in the colonized plants only under salinity stress (p < 0.05; Table 1). Colonization did not alter the stomatal conductance or transpiration rates of the plants in both control and salt stress conditions. Cell membrane stability (CMS), an index of membrane stability was significantly higher in plants colonized by the endophyte compared to plants not treated with the endophyte (Supplementary file 4). However, the chlorophyll stability index (CSI) remained unaltered by endophyte treatment. These results suggest that besides promotion of basic processes associated with growth and development, the fungus stabilizes the cell membrane under salinity stress, while gas exchange is not affected.

**Figure 3:**
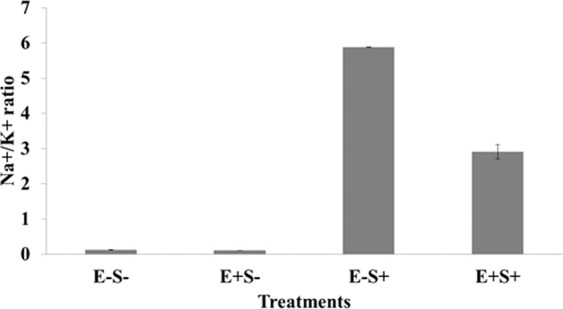
Na^+^/K^+^ ratio of 10 day old IR-64 seedlings subjected to different treatments. Line over bars indicates SEm (±). Legend to X-axis is same as given in Fig. 2A.

To examine the ionic burden of tissues, we examined the Na^+^/K^+^ ratio in plant tissues that were colonized versus those uncolonized by the endophyte. The endophyte-colonized plants had a significantly lower Na^+^/K^+^ ratio (2.9 ± 0.6) than the uncolonized control plants (5.8 ± 0.0) under salinity stress (Fig. 3). The decrease in ratio appears to be primarily driven by a reduced level of tissue Na^+^ in plants colonized by the endophyte compared to those uncolonized. Thus endophyte colonization directly helps in reducing the tissue Na^+^/K^+^ and hence the salt injury. This could indeed be an important mechanism in endophyte mediated mitigation of salt stress in plants.

Intriguingly, while the MDA content of tissue increased significantly, under both, control and salt stress condition, upon colonization by the endophyte, that for proline accumulation decreased upon colonization by the endophyte (Table 1). Finally, we examined the relative SOD activity in tissues of plants colonized and uncolonized by the endophyte. Under both control and salt stress conditions, endophyte did not significantly alter the SOD activity (Table 1).

### Transcriptome sequencing, quality control and reference assembly

To further elucidate the effect of the fungus on the performance of the plants under salt stress, we compared expression profiles of colonized and uncolonized seedlings grown on salt media to identify genes which are targeted by the fungus. A total of 46.31 million reads (9.26 GB; E+S+) and 52.17 million reads (10.43 GB; E−S+) were generated and the raw reads were subjected to basic quality control using in-house Perl scripts and Picard tools v 1.115. The final set comprising of 42.23 million (E+S+) and 47.63 million (E−S+) reads with ≥ Q30 was used for assembly and analysis (Supplementary file 10). The high-quality reads from both treatments were reference-mapped to the *Oryza sativa* Nipponbare genome and 81.50% (E+S+) and 73.25% (E−S+) HQ reads were successfully aligned (Supplementary file 10).

#### Analysis of DGEs and GO enrichment analysis

In seedlings colonized by *Fusarium sp*. and grown under salinity stress, 1348 genes (≥1 log_2_FC) were up-regulated and 1078 genes (≤−1 log_2_FC) down-regulated compared to the uncolonized control (Supplementary files 5 and 9). The DGEs code for proteins involved in both abiotic and biotic stress tolerance such as ion transporters, dehydrogenases, oxidoreductases, as well as transcription factors such as WRKY, MYB, bHLH and bZIP known to be involved in salinity tolerance.

**Figure 4:**
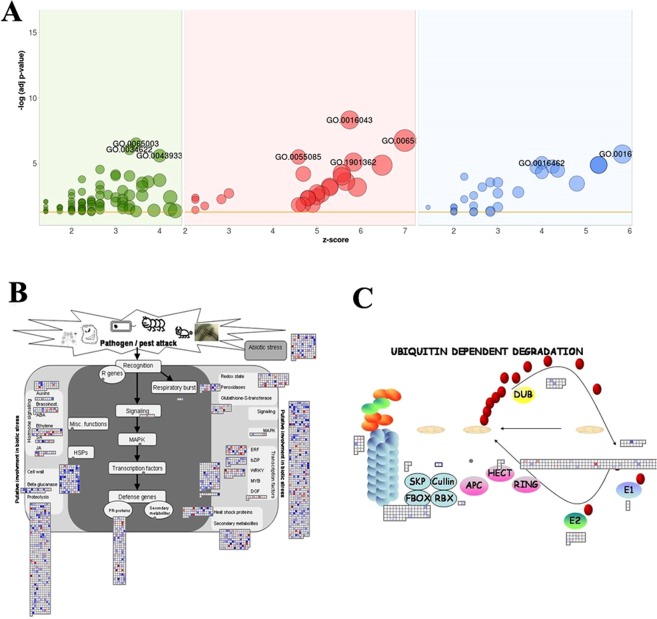
Gene enrichment analysis: (**A**) GO annotation of the significant DGEs. Biological process (green); Cellular component (pink) and Molecular function (blue) (Bubble size is directly proportional to number of genes under the GO term). (**B**) MapMan analysis: MapMan visualization of the DGEs involved in the stress response and (**C**) Ubiquitin-mediated protein degradation pathway as influenced by treatment with endophyte *Fusarium sp*. under salinity stress (Blue pixel: Upregulated genes; Red pixel: down regulated genes).

Among the DGEs, 112 up-regulated genes and 66 down-regulated genes could not be assigned to any known function and hence were classified as transcripts for proteins of unknown function (PUFs). Ten of the top, up- and down-regulated PUFs were computationally annotated for its putative function (Supplementary file 11). Other than the DGEs, a total of 3466 genes were exclusively expressed only in the endophyte enriched transcriptome and were mainly involved in the cell growth promotion and catalytic activity (Supplementary file 5). The results of GO enrichment analysis are shown in Fig. 4A. DGEs were enriched in categories such as transmembrane transport (GO: 0055085) and pyrophosphatase activity (GO: 0016462) (Fig. 4A, Supplementary file 6).

#### MapMan and interaction network analysis

The DGEs were assigned to rice MapMan classification, which covers 36 BINs and its subBINs^19^. The functional pathway classification of the DGEs showed that 576 deduced protein sequences mapped to the ‘overview of metabolism’, with 138 of them belonging to “secondary metabolism”, 203 to “lipid metabolism” and 32 to “redox metabolism” (Supplementary file 7). Further, under ‘overview of cell functions’, 889 deduced protein sequences mapped to the category “transcription regulation”, followed by 667 sequences to “signaling” and 511 to “protein degradation”(Supplementary file 7). Furthermore, stress-responsive transcription factors, such as bHLH, MYB, WRKY, and bZIP proteins, were enriched in the RNA preparation from salt-exposed and endophyte-colonized seedlings. Under the category ‘biotic and abiotic stress response’, the majority of the deduced protein sequences mapped to “signaling” (627 RNAs) and “ubiquitin mediated protein degradation” (511 RNAs) (Fig. 4B). Finally, 288 deduced protein sequences mapped to the category ‘proteosome function’, and within this category, 138 belonged to “ubiquitin E3 RING” and 67 to the “F-box” subgroups (Fig. 4C, Supplementary file 7).

**Figure 5:**
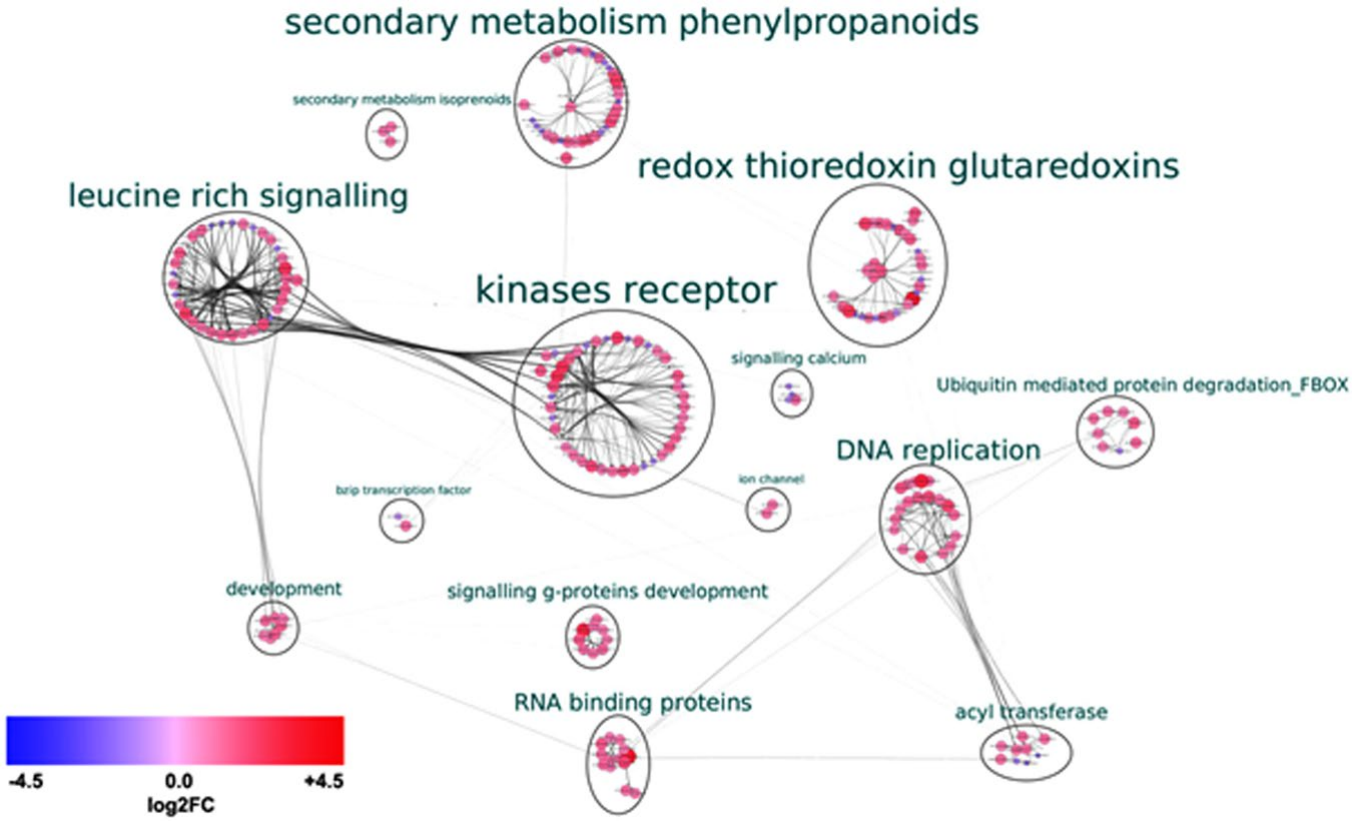
Cytoscape visualization of interaction analysis of the DGEs identified between the two transcriptome libraries: E−S+ and E+S+ (where E: *Fusarium sp*.; S: Salinity stress (150 mM NaCl); +/−: Presence/absence). The clusters have been grouped based on their function and the size indicates the number of genes. The nodes indicate the interaction between the hubs.

To understand the functional relationships and identify major hubs between the proteins encoded by the DGEs, an interaction network analysis was conducted. Altogether, 225 transcripts were predicted to code for proteins that interact with each other. These sequences were further grouped based on their MapMan bins into 14 hubs. Four major hubs contain proteins for signaling through leucine-rich repeat (LRR) proteins, receptor-like kinases (RLKs), secondary metabolism and glutaredoxins. Minor hubs contained proteins involved in calcium signaling, ubiquitin-mediated protein degradation, development, ion channels and transferases (Fig. 5; Supplementary file 8). We also noted substantial crosstalk between the members of the protein groups. For instance, the LRR proteins and RLKs interacted with proteins involved in ion transport and Ca^2+^ signaling, or with F-box proteins of the ubiquitin-mediated protein degradation machinery (Fig. 5, Supplementary file 8). Enzymes involved in the phenylpropanoid pathway are predicted to interact also with those involved in the isoprenoid pathway (Fig. 5).

#### Validation of expression patterns by qPCR analysis

**Figure 6:**
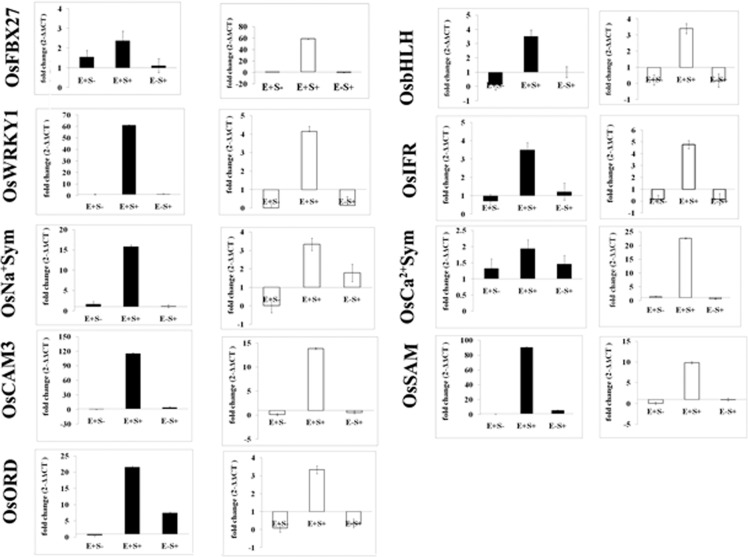
Validation of nine selected DGEs by qPCR. Gene expression levels at E+S−, E+S+ and E−S+ of shoot and root were compared to E−S− and *UBQ10* (Endogenous gene) of shoot and root respectively. Black bars represent shoot and white bars represent root. Errors bars indicate mean ± standard error obtained from biological replicates.

In order to validate the transcriptome data, fifteen genes were chosen (Supplementary file 12). Nine genes which qualified the preliminary screening were further chosen for the qPCR analysis. The analysis was carried out for the four treatments (E−S− (control), E+S−, E+S+, E+S−) on shoot and root tissues separately. The transcript levels of the selected genes were significantly higher in the E+S+ samples compared to the control, in both root and shoot tissues (Figs. 4B and 6).

Interestingly, the transcript levels for OsIFR, OsWRKY1, OsCAM, OsbHLH and OsORD were down-regulated in the roots of seedlings exposed to the E+S− and E−S− treatments, but up-regulated in roots under salinity stress and the presence of *Fusarium sp*. (E+S+). Also, transcripts for OsNa^2+^Sym were only detectable in samples exposed to salinity stress and the endophyte in both shoots and roots (Fig. 6). The qPCR data and the corresponding RNA seq data were significantly positively correlated (r = 0.49, p < 0.05).

## Discussion

It is now becoming abundantly clear that endophytes can effectively modulate plant responses to abiotic stresses and thereby help mitigate the adverse consequences of the stress. A recent meta-analysis conducted using 94 endophyte strains and 42 host plant species from the literature clearly indicates that endophytes promote growth of plants subjected to drought, salinity and nitrogen deficient stresses^20^. In fact if optimized, endophytes could become a potential alternative route towards improving crop performance against abiotic stresses. Set against this background, the results of our study, for the first time demonstrate the successful transfer of a salt tolerant endophyte, from the salt adapted rice, Pokkali, to a salt sensitive rice variety, IR-64 and further on, making the latter salt tolerant.

There has been considerable interest in Pokkali rice and the mechanism underlying its salt tolerance. Li *et al*.^21^ showed that in contrast to the widely grown, but salt-sensitive rice variety IR-29, genes responsible for cell wall integrity, detoxifying ROS and photosynthesis are highly expressed in the salt-tolerant Pokkali rice. Novel rhizosphere-associated bacteria^22–24^ such as *Sphingomonas pokkali*^25^ have been shown to be associated with adaptation of the Pokkali varieties to the brackish rhizospheric environment. We show that the Pokkali races harbor a diversity of endophytic fungi, some of which are extremely saline tolerant. One of the isolates, *Fusarium sp*. is able to thrive even at 2.0 and 2.5 M NaCl. While halophilic fungi have been reported earlier, there are only a few reports on endophytic fungi which are tolerant to such high salt concentrations^26–29^. Besides serving as interesting genetic resources, they could potentially be used in ameliorating salinity stress in non-host plants, as demonstrated in the present study.

### Pokkali endophyte promotes growth of the salt sensitive rice variety, IR-64 under salinity stress

A number of plant growth promoting endophytes have been shown to reduce salt stress in a range of plants including wheat, rice, ryegrass, Arabidopsis and poplar^30–36^. Our results show that the salt-tolerant endophyte *Fusarium sp*. from Pokkali rice successfully colonizes the salt-sensitive variety IR-64 variety and confers salt stress tolerance to the new host. Under salinity stress, the endophyte promotes root and shoot growth of the plants. The tiller number and biomass also increased under salinity stress in the presence of the endophyte. Better performance of the colonized and salt-stress exposed plants was not only evident from higher net assimilation rates and chlorophyll content, but also from a lower Na^+^/K^+^ ratio. Similar results were recently shown by Afridi *et al*.^30^ for wheat and Lanza *et al*.^34^ for *Arabidopsis*. The authors characterized two transporters involved in Na^+^/K^+^ efflux, *SiENA1* and *SiENA5*, and showed that their expression is induced upon exposure to salinity as well as colonization by an endophytic fungus. Consequently, several key stress responses as well as mitigation effects were lowered due to diminished osmotic stress, higher chlorophyll levels and cell membrane stability index, as well as lower proline concentrations. Also Kasotia *et al*.^37^ reported reduced Na^+^/K^+^ ratios in soybean plants inoculated with an *Pseudomonas koreensis* strain and Abdelaziz *et al*.^38^ showed similar effects for Arabidopsis seedlings inoculated with *P. indica*.

### Differential gene expression in the rice variety IR-64 on colonization by Pokkali endophyte

The expression profiles demonstrate that genes related to abiotic and biotic stress tolerance were upregulated under salinity stress in IR-64 plants colonized by the Pokkali endophyte. The upregulated genes include glycosyl hydrolase/chitinases known to act as defense compounds against fungal infestation, ion- and antiporters, dehydrogenases, oxidoreductases, and salinity tolerance-related transcription factors. While some of the genes have been well characterized in other beneficial symbioses^39–41^, early signaling events in mutualistic associations resulting in stress-tolerance responses are not well understood^30,34,36^.

**Figure 7:**
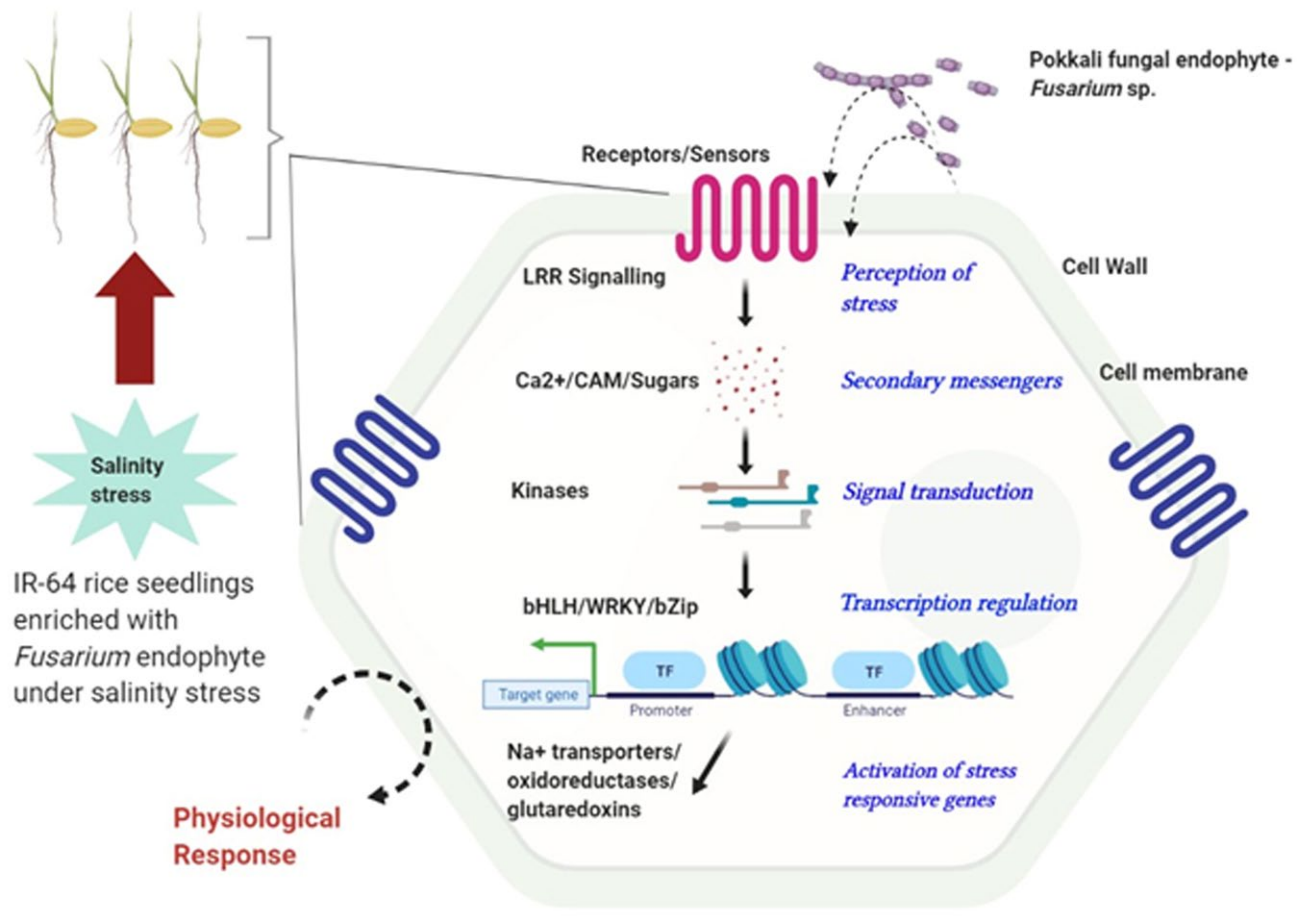
Schematic representation of possible signaling cascades induced by Pokkali endophyte, *Fusarium sp*. in IR-64 plants subjected to salinity stress.

Basically, plant responses to abiotic stresses can be classified into four discrete stages: signal perception, signal transduction, activation of stress-responsive genes, and activation of physiological and metabolic responses^42,43^. Abiotic signal perception, such as salt stress, largely occurs at the cell wall, which activates intracellular signaling including calcium ions, sugars, ROS and phosphorylation events, followed by downstream responses^42,44,45^. In one of the comparative transcriptome studies, involving a transgenic ‘Khao Dawk Mali 105’ rice over-expressing *OsCam1–1*, it was demonstrated that the overexpression altered the expression of several genes involved in cellular processes under salt stress, such as signaling, lipid, carbohydrate, secondary metabolism, photosynthesis and others^46^. In the present study, we found that the Pokkali endophyte triggered the expression of Ca^2+^ ion signaling which further enhanced the expression of downstream stream response genes. These were reflected in the DGEs and qPCR data which uncovered genes involved in many of these processes (Supplementary file 5; Figs. 2, 7, Table 1).

The interaction network analysis of the proteins encoded by the DGEs identified components involved in signaling through LRR proteins and receptor kinases that play a major role in external signal perception. Their specificity in salt stress tolerance responses need to be elucidated although it has been shown that they are involved in various environmental stress responses^47^. In a study on sorghum, it was demonstrated that the receptor kinases genes were upregulated under salt stress^48^. Also transgenic plants overexpressing a *GbRLK* gene modulated the expression of genes implicated to have roles in both, biotic and abiotic stresses^49,50^.

The qPCR analyses showed that genes involved in calcium signaling and transport were highly up regulated by the Pokkali endophyte in both, roots and shoots. Ca^2+^ and calmodulin activate cascades of NaCl – inducible genes, which in turn bind to the E-box element (CANNTG) in the promoter regions of several salt stress-responsive genes or activate R2R3-type MYB2 transcription factors of salt stress-responsive genes^51^. In a study involving different rice varieties under salt stress, it was demonstrated that Ca^2+^ plays an important role in root to shoot signaling leading to the exclusion of sodium from the shoots^52^. A number of candidate genes for this scenario are given in Supplementary file 5. A WRKY transcription factor was found to be up-regulated in IR-64 plants colonized by the Pokkali endophyte (Supplementary file 5; Fig. 6). In poplar, Shen *et al*.^53^ demonstrated that over-expression of *PeWRKY1* lead to enhanced salinity tolerance and better root growth, photosynthesis, survival rates under stress, and ion fluxes.

Besides specific salt stress-responsive transcription factors (e.g. Todaka *et al*.^54^), genes for ‘ubiquitin mediated protein degradation’ were also induced by endophyte colonization. The ubiquitin–proteasome system mediates protein degradation, but also modulates the localization, activity and stability of proteins and is an important stress regulator since it functions across numerous signaling pathways^55^. The identified F-box genes are part of a large gene family in plants and are involved in degradation of cellular proteins through ubiquitin-mediated degradation. A recent study revealed that over-expression of a wheat F-box gene induces salinity tolerance in transgenic tobacco^56^.

The low Na^+^/K^+^ ratio in plants treated with the endophyte (Fig. 3) involves ion transporters, though as yet, we do not know which of the identified transporters are directly involved in the salt tolerance response. Comparison of our data with those obtained for halophytic turf grass, *Sporobolus virginicus*^57^, might help to identify crucial genes involved in the process.

Many of the upregulated genes also code for enzymes involved in secondary metabolism. While they are believed to be associated with biotic stresses, they are also expressed in response to abiotic stresses^16,17^, as shown in the transcriptome analysis by Chandran *et al*.^58^ for a japonica rice variety under salinity stress. Glycosylated flavonoids, hydroxycinnamic acids and chlorogenic acid are involved in antioxidative activity and induced in plants following endophyte colonization^59^. Another major hub unraveled by the interaction network was the antioxidants ‘glutaredoxins’. In rice, the glutaredoxin 20 functioned as a positive regulator in tolerance to multiple abiotic stresses^60^.

In summary, our study demonstrates that a salt tolerant endophyte, *Fusarium sp*., from the salt-adapted Pokkali rice, can be successfully transferred to the cultivated salt-sensitive rice variety, IR-64 to confer salt tolerance. The agricultural importance of this finding will stimulate future investigations on the molecular basis of this beneficial symbiotic interaction as also allowing for developing applications in real world agricultural situations.

## Materials and Methods

### Plant material

Seeds of salt-adapted Pokkali rice (*Oryza sativa* L.) land races, namely, VTL-4, VTL-6 and VTL-8^61^ were obtained from the Rice Research Station, Vytilla, Kerala Agricultural University, Kerala, India (9.977920° N–76.319530°E) and salt-sensitive varieties (IR-64 and JBT 36/14)^62^ from the Department of Crop Physiology, University of Agricultural Sciences, GKVK, Bengaluru, India (12.946550°N–77.509850°E).

### Isolation of endophytic fungi

Endophytic fungi were isolated from the three Pokkali varieties and the salt sensitive rice varieties, IR-64 and JBT 36/14. Pre-germinated seeds of these varieties were raised in plastic pots measuring 14 ×14 ×16 cm containing medium “red loam soil + farm yard manure”. In each pot, six seedlings were raised. The pots were regularly watered. Twenty-five days after planting, explants from roots, shoots and seeds were used for isolation of endophytic fungi. The explants (1 cm long) were surface-sterilized as described by Arnold *et al*.^63^. The tissues were blot-dried and transferred to Petri plates containing potato dextrose agar (PDA) and incubated at 25 ± 2 °C for 10 days under 12 hour photoperiod for emergence of endophytic fungi^64^. Imprints of sterilized cut segments were examined on PDA plates for the effectiveness of surface sterilization procedure^65^. The frequency of colonization by endophytic fungi was determined as described previously^66^. Fungi that emerged from the cut ends of the tissue were sub-cultured onto fresh PDA plates to obtain pure cultures. The purified isolates were cultured on PDA slants and stored at 4 °C.Voucher numbers were assigned to each of the isolates and deposited in the School of Ecology and Conservation Lab, University of Agricultural Sciences, GKVK, Bengaluru, India.

### Morphological and molecular characterization of fungal endophytes

The purified endophytic fungal isolates were classified into operational taxonomic units (OTUs) based on the culture characteristics and morphology of reproductive structures/spores/conidia^67–72^.

For the molecular characterization, genomic DNA was extracted from the fungal mycelium using the cetyl trimethyl ammonium bromide (CTAB) method^73^. The universal internal transcribed spacer (ITS) primers, ITS1 and ITS4^74^ were used to amplify fungal *ITS* regions (ITS1-5.8S- ITS2). Amplified products were quantified and sequenced by Shrimpex Biosciences, Chennai, India. Further, the obtained sequences were homology-searched using NCBI BLAST program to confirm the obtained sequence as ITS1-5.8S-ITS2 (http://blast.ncbi.nlm.nih.gov; default parameters). The identification of the endophyte was ascertained based on maximum query coverage and score in the BLAST results^75^.

### Evaluation of endophytic fungal isolates for salinity stress tolerance

Five-day-old test fungal isolates (culture disc of 5 mm diameter) were placed aseptically on PDA plates containing different NaCl concentrations (200 mM, 400 mM, 600 mM, 800 mM and 1000 mM) and control (without NaCl) in triplicate. The plates were incubated at 25 ± 2 °C for 5 day under 12-hour photoperiod. Mycelial growth was recorded on the 5^th^ day after inoculation. Percent reduction in mycelial growth (measured in mm) over the respective controls was calculated following Bekker *et al*.^76^. The fungal endophytes that showed little reduction (ranging from 0 to 20 per cent) in 1 M NaCl were further evaluated for their tolerance at 1.5 M and 2 M NaCl. Based on the above screening protocol, we identified one endophytic fungus, *Fusarium sp* (GenBank Acc No. MN170565) obtained from the Pokkali variety VTL-4, as salt tolerant (Fig. 1A,B). We conducted all further experiments using this endophyte.

### Evaluation of the endophyte, *Fusarium sp* (GenBank Acc No. MN170565), for its ability to impart salinity tolerance to the salt sensitive rice variety, IR-64

The endophyte *Fusarium sp*. **(**GenBank Acc No. MN170565) was evaluated for its ability to impart salinity tolerance to a salt-sensitive rice variety IR-64. The seeds of IR-64 were surface-sterilized according to Arnold *et al*.^63^ and germinated for 48 hours. Five-day-old fungal colony cultures were used for inoculum preparation by washing the mycelial mat with sterile distilled water. Pre-germinated seeds were treated with the mycelia suspension (2 ×10^6^ cfu/ml) for 3 hours along with control (treated with sterile distilled water). The seedlings were then transferred to moistened germination sheet and paper towels^9^. One set of the paper towels was treated with 150 mM NaCl and incubated at 28 ± 2 °C for 7 days. Another set was treated with sterile distilled water and maintained as control. The colonization ability of *Fusarium* sp. was determined in 10-day-old seedlings by re-isolation of the endophyte following the method described above. The tissue generated in the experiment was used to assess the morphological, biochemical and molecular parameters of the host plant.

To further evaluate the ability of *Fusarium* sp. to confer salt tolerance to IR-64, pot experiments were carried out in the greenhouse using non-autoclaved soil. Ten-day-old IR-64 seedlings (pre-treated with *Fusarium* sp. or non-treated) were transferred to plastic pots (14 ×14 ×16 cm) and grown in a potting medium (red loam soil + farm yard manure). The experiment was carried out in three biological replications. In each pot, six seedlings were transplanted and then subjected to salinity stress (4 ds/m) for fifty days^77^. The composition of salts used for imposing salinity stress (4 ds/m**)** was 1463 mg/l of calcium chloride dehydrate (CaCl_2_.2H_2_O), 459 mg/l of sodium chloride (NaCl), 381 mg/l of magnesium sulphate (MgS0_4_.7H_2_0), 1849 mg/l of magnesium chloride, hexahydrate (MgCl_2_.6H_2_0). Control sets of rice seedlings were irrigated with sterile water.

#### Plant growth and tiller number

Several growth parameters were recorded on both the 10-day old seedlings (raised on paper towels) and 50-day old plants raised in plastic pots for the four treatments E+S−; E−/S−; E+S+ and E−/S+, where E+/E− refers to presence/absence of endophyte and S+/S− refers to presence /absence of salinity stress. Growth, as well as root and shoot lengths were recorded for 10-day old seedlings. Plant height, tiller number and total biomass were recorded for 50-day old plants.

#### Gas exchange parameters, chlorophyll content, chlorophyll stability index, cell membrane stability and Na^+^/K^+^ ratio

Carbon assimilation (A), stomatal conductance (gs) and rate of transpiration (T) were recorded in the flag leaf of 50-day old IR-64 plants exposed to the four treatments (E−S−; E+/S−; E+S+; E−/S+) using an Infrared Gas analyzer-IRGA (LiCOR C6400, Inc. Lincoln, Nebraska, USA).

The total chlorophyll content, chlorophyll stability index (CSI) and cell membrane stability (CMS) was estimated in 10-day old IR-64 seedlings. Chlorophyll was extracted and determined using the UV visible spectrophotometer from Shimadzu (Japan) at 663 and 645 nm according to Hiscox and Israelstam^78^ and the amounts of chlorophyll *a* and *b* were calculated following Arnon^79^. The CSI was calculated using the formula: CSI = 100-CSI%, where CSI% is percent reduction of chlorophyll under salinity stress over that of the non-stress-exposed control.

The cell membrane stability (CMS) test was carried out according to the method described by Blum and Ebercon^80^. The initial (T1) and final electrolyte leakage (T2) after incubating the tissues in boiling water for 30 mins was determined as described by Tripathy and co-workers^81^ using a conductivity meter, EC-TDS (Elico-CM183). The CMS was calculated as described in Blum and Ebercon^80^.

The Na^+^ and K^+^ contents were analyzed following wet digestion with diacid (nitric acid: per chloric acid) (10:4 v/v) as outlined by US Salinity Laboratory Staff (1954)^40^, using an Inductively Coupled Plasma Optical Emission Spectrophotometer (ICP-OES, Thermo Scientific Inc, USA). Three replicates were maintained for all the experiments described above.

#### Superoxide dismutase activity, malondialdehyde and proline content

Superoxide dismutase (SOD) activity, malondialdehyde (MDA) and proline content were estimated in the 10-day old IR-64 seedling for the four treatments. SOD activity was measured as described previously at 560 nm based on the reduction of nitro-blue tetrazolium (NBT)^82^. One unit of SOD activity was expressed as the amount of enzyme utilized to inhibit 50% of NBT reduction. The MDA content was measured by the thiobarbutaric acid (TBA) method^83^. The proline content was determined by the acid ninhydrin method^84^ and the amount in the tissue calculated as described by Khaliel *et al*.^85^.

### Molecular characterization

#### RNA extraction, library preparation and Illumina HiSeq 2500 sequencing

The root and shoot tissues of 10-day old IR-64 seedlings (E−S+ and E+S+) were frozen in liquid nitrogen and ground to obtain fine powder. The RNA was extracted using RNAisoPlus following the manufacturer’s protocol (Takara). Equal amounts of RNA from root and shoot were pooled from 3 independent experiments, each of which consisted of RNA from 75 seedlings, for the library preparation. The messenger RNA isolation with polyA selection and subsequent library construction with the TruSeq RNA sample preparation protocol from Illumina (San Diego, CA) was carried out. Paired end (PE) sequencing was performed on the Illumina HiSeq. 2500 sequencing platform (SciGenom Labs Pvt Ltd, Cochin, Kerala, India). The raw data has been deposited at NCBI (PRJNA511618 and PRJNA511516).

#### Reference assembly and DGE analyses

The high quality Paired End reads from E−S+ and E+S+ treatments were aligned independently to the *Oryza sativa* Nipponbare genome (rice.plantbiology.msu.edu/pub/data/Eukaryotic_Projects/o_sativa/annotation_dbs/pseudomolecules/version_7.0/all.dir) using the software TopHat v 2.1.0^86^. The bam file generated was further used to obtain the readCount file using the featureCount program (http://subread.sourceforge.net)^87^. Filtering and normalization of the reads within and between the treatments were performed using the “cpm” and “calcNormFactors” functions of the EdgeR software. Further, biological coefficient of variation (BCV) was calculated using the “estimateGLMCommonDisp” function. Finally, differential expression p-values were computed using the readCount files of the two treatments using the EdgeR “ExactTest” method (https://bioconductor.org/packages/release/bioc/html/edgeR.html)^88^ which can handle data with small sample size^89^. The “ExactTest” method is the quantile-adjusted conditional maximum likelihood (qCML) method, which is applicable for single factor analysis. It also has been shown to have a strong parallel with the Fisher’s exact test^88^. Thus, edgeR’s “Exact Test” method gives more stable and biologically meaningful gene rankings, especially when dealing with pooled samples, as had been conducted in our study. The volcano plot was drawn in R. The analysis revealed that there were many genes for proteins of unknown functions (PUFs). The top 10 up- and down-regulated genes were annotated for its putative functions using the web server tool PUFAS (http://caps.ncbs.res.in/pufas/)^90^.

#### Gene ontology enrichment and mapMan analysis

Gene Ontology (GO) analysis was performed on DGEs (≥1 log_2_FC and ≤−1 log_2_FC) using the online tool AgriGO v 2.0 (systemsbiology.cau.edu.cn/agriGOv2/)^91^. The GO terms were visualized using the R package GOPlot^92^.

The MapMan program v 3.5.1 was used for the pathway analysis (https://mapman.gabipd.org/home)^93^. The pathway mapping of *Oryza sativa* Nipponbare (Osa_MSU_v7) were downloaded from the MapMan store (https://mapman.gabipd.org/mapmanstore). The non-redundant genes from edgeR (≥1 log_2_FC and ≤−1 log_2_FC) were then classified into MapMan BINs, and their annotated functions were visualized using the Osa_MSU_v7 pathway annotation database of the MapMan program.

#### Interaction network of the proteins encoded by the DGEs

To determine the interactions of proteins from the DGEs (≥1 log_2_FC and ≤−1 log_2_FC), Protein Protein Interaction (PPI) analysis was performed using the online tool STRING (http://string-db.org)^94^. The PPI network of all proteins of the DGEs was extracted from the whole interaction network and reconstructed using Cytoscape v 3.6.1^95^. The raw network was clustered using the “MCL cluster” algorithm under the cluster maker section. The clustered networks were further annotated using the Auto annotate option using the MapMan bin names. The nodes were color-coded according to their log_2_FC values. Finally, all the edges were bundled using the “bundle edge” option.

#### Validation by qPCR analysis

Root and shoot tissues of 10-day old IR-64 seedlings exposed to one of the four treatments (E−S−; E+S−; E−S+; E+S+) were used for qPCR validation in three biological replications. The RNA was extracted using RNAisoPlus following the manufacturer’s protocol (Takara). The quality of the RNA was analyzed using a Nanodrop spectrophotometer and integrity was checked using agarose gel electrophoresis. Further, DNase treatment (Invitrogen, Carlsbad, CA, USA, AM-1907) and cDNA synthesis (Thermo Scientific, Waltham, MA, USA, #K1621) was carried out according to the manufacturer’s instructions. A total of fifteen genes were chosen based on the bioinformatic analysis of the transcriptome data. Based on the preliminary screening standard (slope −3.3; efficiency 100% ± 4) nine genes were chosen for relative quantification. The reactions were performed in 96 well plates in 20 µl containing 10 µl KAPA SYBR FAST qPCR Master Mix (Kapa Biosystems), 3 pmolml^−1^forward primer, 3 pmolml^−1^reverse primer, 4 µl nuclease-free water and 2 µl cDNA (1: 5 diluted) in an ABI Quant Studio 7 Flex (Applied Biosystems, Foster City, CA, USA). Thermal cycling conditions were 40 cycles of 95 °C for 5 s, 55–59 °C (depending on the primer combinations) for 40 s, and 72 °C for 35 s followed by dissociation curve. The control gene used in the study was *OsUBQ10*^96^. The relative expression was calculated using the equation 2^−ΔΔCT^, where −ΔΔCT = ΔCT − *C*t of the control (E−S−); ΔCT = (*C*t of the target gene − *C*t of the control gene). The list of primers used for amplification is presented in Supplementary file 12.

## Supplementary information

Supplementary information.

Supplementary information2.

Supplementary information3.

Supplementary information4.

Supplementary information5.

Supplementary information6.

Supplementary information7.

Supplementary information8.

Supplementary information9.

Supplementary information10.

Supplementary information11.

Supplementary information12.

## Data Availability

The pooled transcriptome data of IR-64 seedlings under the treatments: E−S+ and E+S+ used in the present study has been submitted to NCBI (SRA) with the accession number PRJNA511618 and PRJNA511516 respectively.
