# Supplementary material for: An endophyte from salt-adapted Pokkali rice confers salt-tolerance to a salt-sensitive rice variety and targets a unique pattern of genes in its new host
Source: Sci Rep. 2020 Feb 24;10:3237. doi: 10.1038/s41598-020-59998-x (PMC7039991; doi:10.1038/s41598-020-59998-x)
Supplement: Supplementary file 1 — Supplementary information. [file 41598_2020_59998_MOESM1_ESM.docx]

**Table S1** Characterization of endophytic fungal OTUs (based on the fungal colony characteristics and color) obtained from salt adapted and sensitive genotypes

| **OTU** | **Colony characteristics and fungal colour** |  | **Number of fungal isolates emerged** | | | | |
| --- | --- | --- | --- | --- | --- | --- | --- |
|  |  | **Control** | **NaCl** | | | | **Total** |
|  |  |  | **100mM** | **150mM** | | **200mM** |  |
| Paddy genotype: VTL-4 | | | | | | | |
| V-4 A | White, pink background | 15 | 16 | 8 | | 0 | 39 |
| V-4 B | White, orange background | 6 | 6 | 4 | | 8 | 24 |
| V-4 C | Pale orange | 1 | 0 | 0 | | 2 | 3 |
| V-4 D | Black | 0 | 2 | 0 | | 0 | 2 |
| V-4 E | Bright white | 1 | 0 | 6 | | 0 | 7 |
| V-4 F | Dark black | 0 | 0 | 1 | | 0 | 1 |
| V-4 G | Pale white | 0 | 0 | 0 | | 0 | 0 |
| V-4 H | White, black background | 0 | 0 | 0 | | 1 | 1 |
| V-4 I | White, dark background | 0 | 0 | 0 | | 2 | 2 |
| V-4 J | Feathery white | 0 | 0 | 0 | | 2 | 2 |
| Paddy genotype: VTL-6 | | | | | | | |
| V-6 A | White with orange background | 23 | 4 | 7 | | 7 | 41 |
| V-6 B | White with pink background | 20 | 19 | 6 | | 11 | 56 |
| V-6 C | White cottony growth | 3 | 6 | 0 | | 0 | 9 |
| V-6 D | Gray | 0 | 2 | 0 | | 0 | 2 |
| V-6 E | Dark orange | 0 | 0 | 0 | | 1 | 1 |
| Paddy genotype: VTL-8 | | | | | | | |
| V-8 A | White with orange background | 13 | 21 | 4 | | 4 | 42 |
| V-8 B | White with pink background | 9 | 0 | 14 | | 8 | 31 |
| V-8 C | Whitish growth | 2 | 0 | 0 | | 0 | 2 |
| V-8 D | Black | 6 | 0 | 0 | | 0 | 6 |
| V-8 E | Black with white dots | 0 | 5 | 0 | | 0 | 5 |
| V-8 F | Dark black | 0 | 2 | 4 | | 0 | 6 |
| V-8 G | Pale white | 0 | 0 | 0 | | 6 | 6 |
| Paddy genotype: IR -64 | | | | | | | |
| IR A | White with orange | 21 | 0 | | 0 | 5 | 26 |
| IR B | White with pink | 9 | 0 | | 0 | 0 | 9 |
| IR C | Dark black | 3 | 5 | | 0 | 0 | 8 |
| IR D | Dark with spots | 3 | 2 | | 0 | 6 | 11 |
| IR E | Grayish | 2 | 0 | | 0 | 0 | 2 |
| IR F | Dark background | 1 | 0 | | 2 | 2 | 5 |
| IR G | White with yellow | 0 | 6 | | 9 | 0 | 15 |
| IR H | White with gray | 0 | 1 | | 0 | 0 | 1 |
| IR I | Light dark | 0 | 2 | | 0 | 0 | 2 |
| IR J | Feathery ash | 0 | 1 | | 0 | 0 | 1 |
| IR K | White dotted | 0 | 2 | | 0 | 0 | 2 |
| Paddy genotype : JBT 36/14 | | | | | | | |
| JBT A | White with pale yellow | 9 | 0 | | 0 | 0 | 9 |
| JBT B | Gray | 7 | 0 | | 0 | 0 | 7 |
| JBT C | Orange | 3 | 0 | | 0 | 0 | 3 |
| JBT D | Feathery white | 4 | 0 | | 0 | 0 | 4 |
| JBT E | Flat gray | 0 | 18 | | 7 | 0 | 25 |
| JBT F | Dark black | 0 | 7 | | 6 | 0 | 13 |
| JBT G | Pale white | 0 | 6 | | 3 | 4 | 13 |
| JBT H | Dark Gray | 0 | 0 | | 2 | 4 | 6 |

**Table S2:** Total number of endophytes obtained from salt adapted and sensitive genotypes and their colonization frequency.

| **Paddy genotype** | **Type** | **Plant Parts** | **Explants** | **Number of**  **endophytes**  **emerged** | **Colonization frequency (%)** |
| --- | --- | --- | --- | --- | --- |
| VTL-4 | Salt adapted  Pokkali | Shoot | 15 | 7 | 46.6 |
|  |  | Root | 45 | 24 | 53.3 |
|  |  | Seed | 60 | 56 | 93.3 |
| VTL-6 | Salt adapted  Pokkali | Shoot | 30 | 29 | 96.6 |
|  |  | Root | 60 | 35 | 58.3 |
|  |  | Seed | 60 | 57 | 95 |
| VTL-8 | Salt adapted  Pokkali | Shoot | 45 | 24 | 53.3 |
|  |  | Root | 60 | 32 | 53.3 |
|  |  | Seed | 60 | 47 | 78.3 |
| IR-64 | Salt sensitive | Shoot | 30 | 21 | 70 |
|  |  | Root | 60 | 35 | 58.3 |
|  |  | Seed | 60 | 45 | 75 |
| JBT36/14 | Salt sensitive | Shoot | 30 | 21 | 70 |
|  |  | Root | 45 | 22 | 48 |
|  |  | Seed | 60 | 39 | 65 |
