# Supplementary material for: An endophyte from salt-adapted Pokkali rice confers salt-tolerance to a salt-sensitive rice variety and targets a unique pattern of genes in its new host
Source: Sci Rep. 2020 Feb 24;10:3237. doi: 10.1038/s41598-020-59998-x (PMC7039991; doi:10.1038/s41598-020-59998-x)
Supplement: Supplementary file 2 — Supplementary information2. [file 41598_2020_59998_MOESM2_ESM.docx]

| **OTU**  **Code** | **Sequence**  **length (bp)** | **Blast search result** | **Query cover (%)** | **Identity (%)** | **Homologue** |
| --- | --- | --- | --- | --- | --- |
| Paddy genotype: VTL-4 | | | | | |
| V-4 A | 909 | *Gibberella intermidia* strain | 95 | 93 | HQ443247.1 |
| V-4 B | 943 | *Arthobotys follicola* | 94 | 99 | U51954.1 |
| V-4 C | 886 | *Gibberella intermidia* strain | 94 | 94 | HQ443247.1 |
| V-4 D | 904 | *Gibberella intermidia* strain | 95 | 94 | HQ443247.1 |
| V-4 E | 907 | *Botryosphaeria dothidea* strain | 88 | 92 | KF938899.1 |
| V-4 G | 885 | *Fusarium solani* strain | 93 | 92 | JX535014.1 |
| V-4 H | 902 | *Arthobotys follicola* | 90 | 99 | U51954.1 |
| V-4 J | 890 | *Fusarium sp.* | 99 | 100 | MH910492.1 |
| Paddy genotype: VTL-6 | | | | | |
| V-6 A | 849 | *Gibberella intermidia* strain | 90 | 93 | HQ443247.1 |
| V-6 B | 865 | *Gibberella intermidia* strain | 90 | 92 | HQ443247.1 |
| V-6 D | 927 | *Pleosporales* species | 98 | 92 | JQ619818.1 |
| V-6 E | 494 | *Fusarium sp.* NSC-12 | 96 | 100 | GU257905.1 |
| Paddy genotype: VTL-8 | | | | | |
| V-8 A | 846 | *Gibberella intermidia* strain | 89 | 93 | HQ443247.1 |
| V-8 B | 879 | *Gibberella intermidia* strain | 93 | 93 | HQ443247.1 |
| V-8 C | 881 | *Botryosphaeria dothidea* strain | 95 | 93 | KF938899.1 |
| V-8 D | 910 | *Alternaria alternate* | 98 | 95 | JQ080319 .1 |
| V-8 F | 891 | *Cochilobolus* | 86 | 100 | KC315940.1 |
| V-8 G | 820 | *Gibberella intermidia* strain | 88 | 94 | HQ443247.1 |
| Paddy genotype: IR-64 | | | | | |
| IR-A | 901 | *Gibberella intermidia* strain | 91 | 94 | HQ443247.1 |
| IR-C | 885 | *Gibberella intermidia* strain | 96 | 92 | HQ443247.1 |
| IR-D | 812 | *Fungal endophyte* strain | 88 | 97 | FJ449944.1 |
| IR-E | 859 | *Gibberella intermidia* strain | 91 | 94 | HQ443247.1 |
| IR-F | 803 | *Cochilobolus miyabeanus* strain | 87 | 99 | KC315919.1 |
| IR-G | 851 | *Fusarium solani* strain | 88 | 93 | JX435014.1 |
| IR-H | 853 | *Fusarium of solani* | 85 | 99 | JX435221.1 |
| IR-I | 881 | *Fusarium of solani* | 92 | 99 | JX435221.1 |
| IR-K | 886 | *Alternaria podowickii* isolate | 91 | 92 | JQ907483.1 |
| Paddy genotype: JBT 36/14 | | | | | |
| JBT A | 914 | *Fusarium solani* strain | 91 | 92 | JX535014.1 |
| JBT B | 874 | *Arthrobotys follicola* | 72 | 89 | U51954.1 |
| JBT C | 912 | *Coniothyrium aleuritis* isolate | 98 | 93 | EU622266.1 |
| JBT D | 901 | *Arthrinium* species | 85 | 98 | KC806224.1 |
| JBT E | 921 | *Pleosporalis* species | 98 | 92 | JQ619818.1 |
| JBT G | 639 | *Fusarium oxysporum* | 19 | 81 | KF907243.1 |
| JBT H | 669 | *Gibberella Intermidia* strain | 99 | 94 | HQ443247.1 |

**Table S2** Molecular characterization of endophytic fungi obtained from salt adapted and sensitive genotypes
