# Supplementary material for: An endophyte from salt-adapted Pokkali rice confers salt-tolerance to a salt-sensitive rice variety and targets a unique pattern of genes in its new host
Source: Sci Rep. 2020 Feb 24;10:3237. doi: 10.1038/s41598-020-59998-x (PMC7039991; doi:10.1038/s41598-020-59998-x)

**Figure S1:** Confirmation of *Fusarium sp.* colonization from paddy (IR-64) tissue segments.


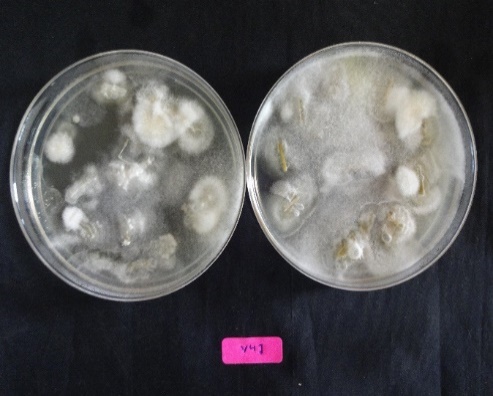


**Figure S2 :** Growth of salt tolerant endophytic fungi, *Fusarium sp.* and salt sensitive endophyte, *Arthrinium* sp. at different NaCl concentrations. Growth of *Fusarium sp.* (1) and *Arthrinium* sp. (2) at control (a), 1 M NaCl (b), 1.5 M NaCl (c) and 2 M NaCl (d) concentrations.


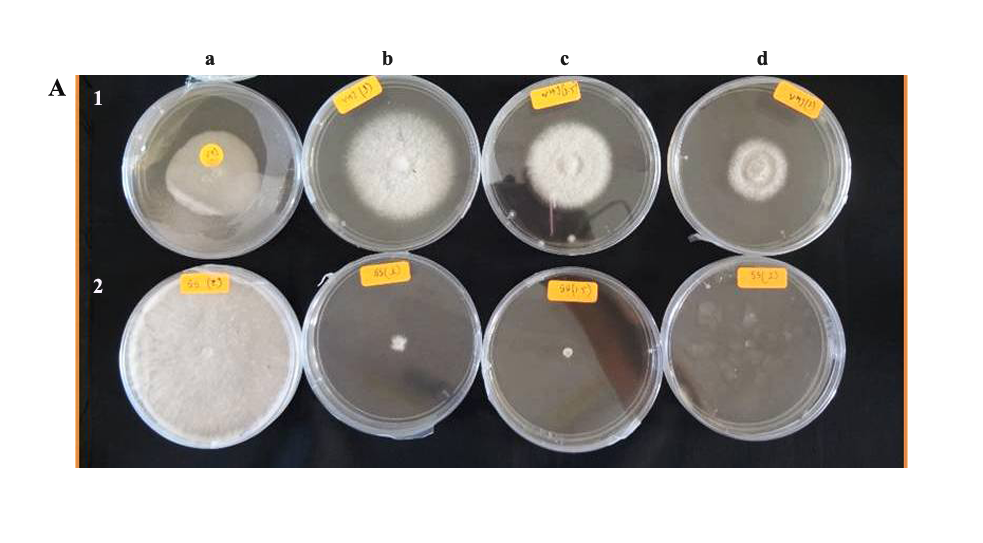


**Figure S3:** Microscopic characterization of the *Fusarium sp*. (V4J). Arrow: Spore of the *Fusarium sp.*


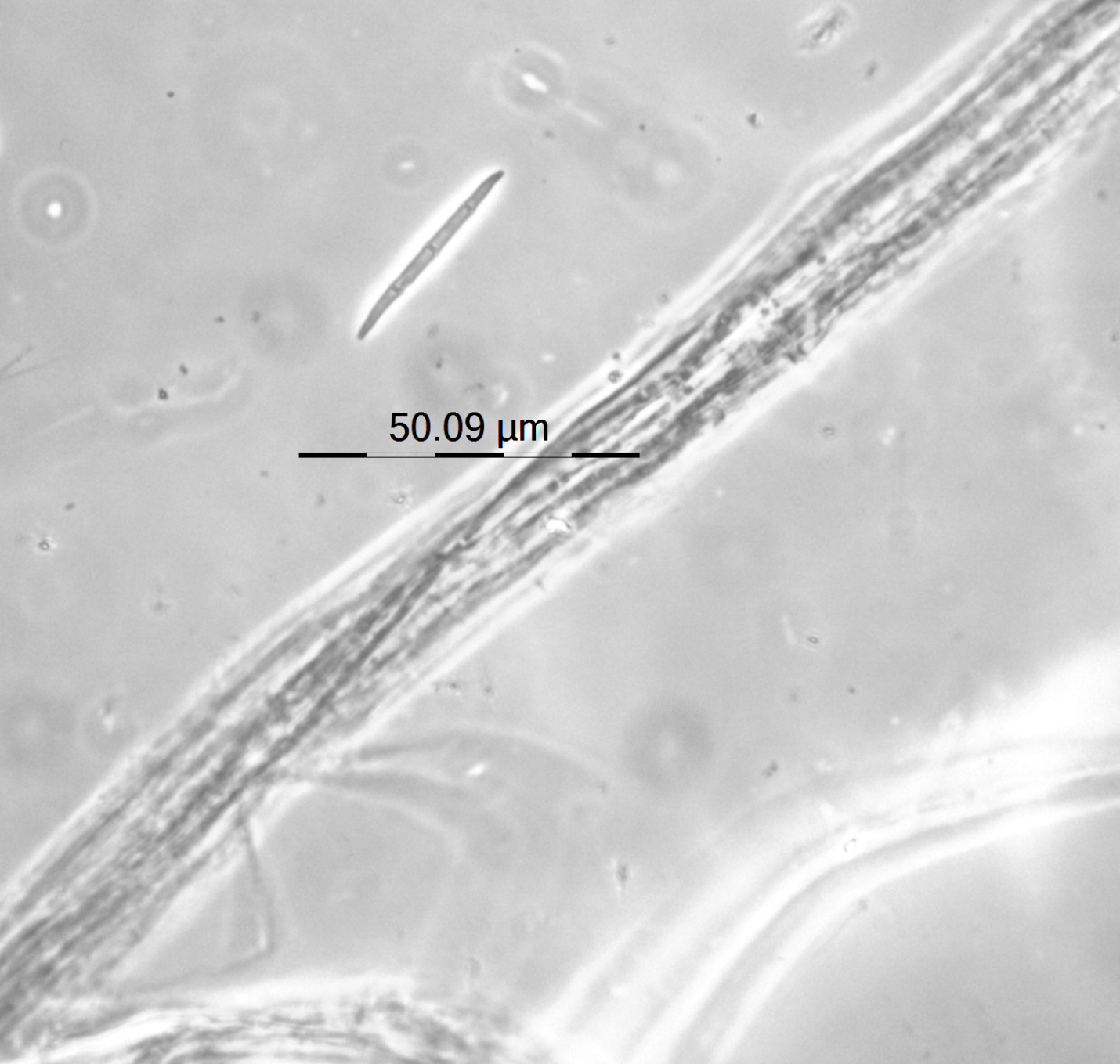

Supplement: Supplementary file 3 — Supplementary information3. [file 41598_2020_59998_MOESM3_ESM.docx]
