# Supplementary material for: An endophyte from salt-adapted Pokkali rice confers salt-tolerance to a salt-sensitive rice variety and targets a unique pattern of genes in its new host
Source: Sci Rep. 2020 Feb 24;10:3237. doi: 10.1038/s41598-020-59998-x (PMC7039991; doi:10.1038/s41598-020-59998-x)

**Figure S4:** Physiological response of IR64 seedling under different treatments (E+/E-: Presence/absence of *Fusarium sp.* ; S+/S- : Presence/ absence of salinity stress) A) Cell membrane stability , B) Chlorophyll stability index.


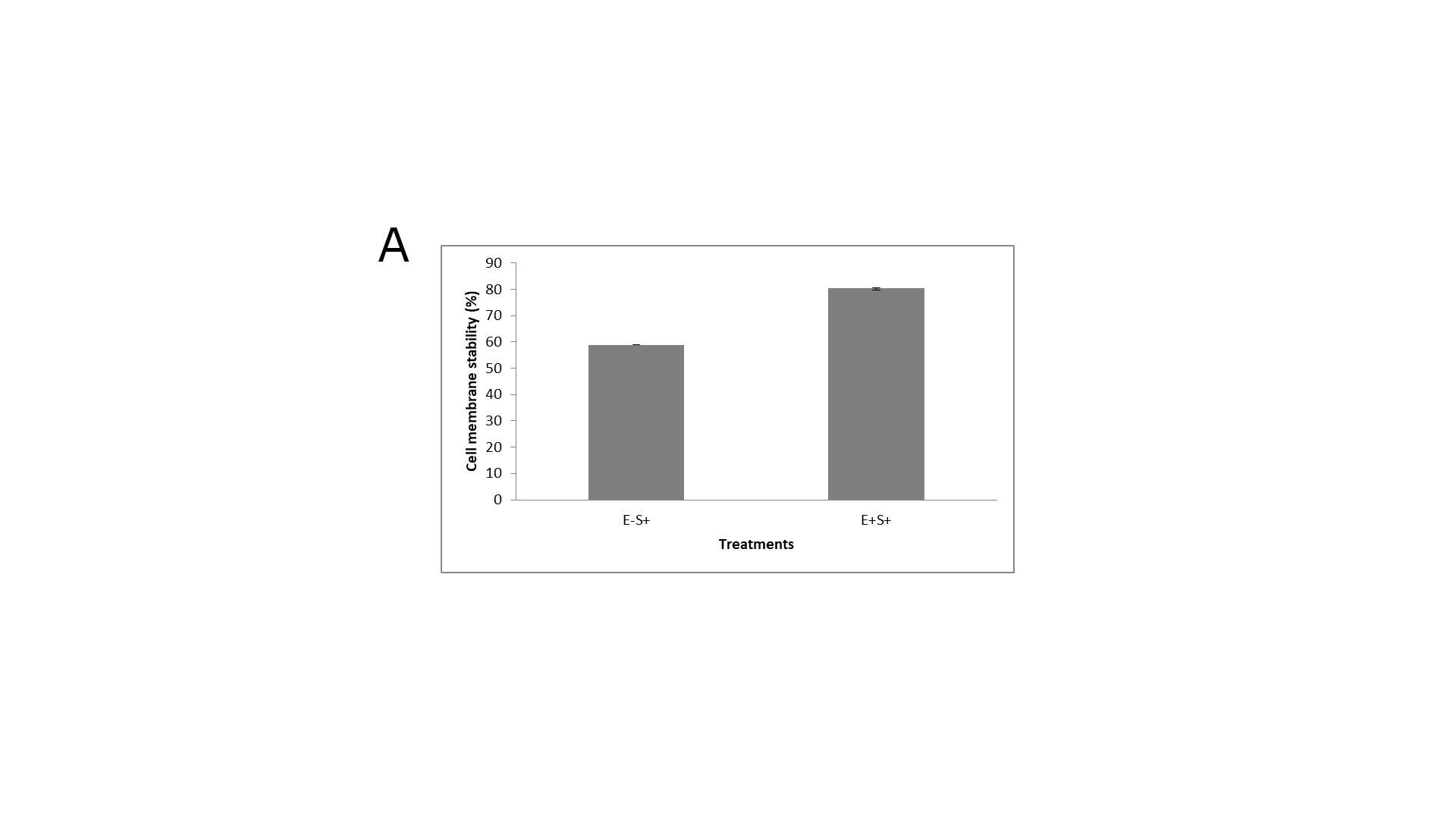


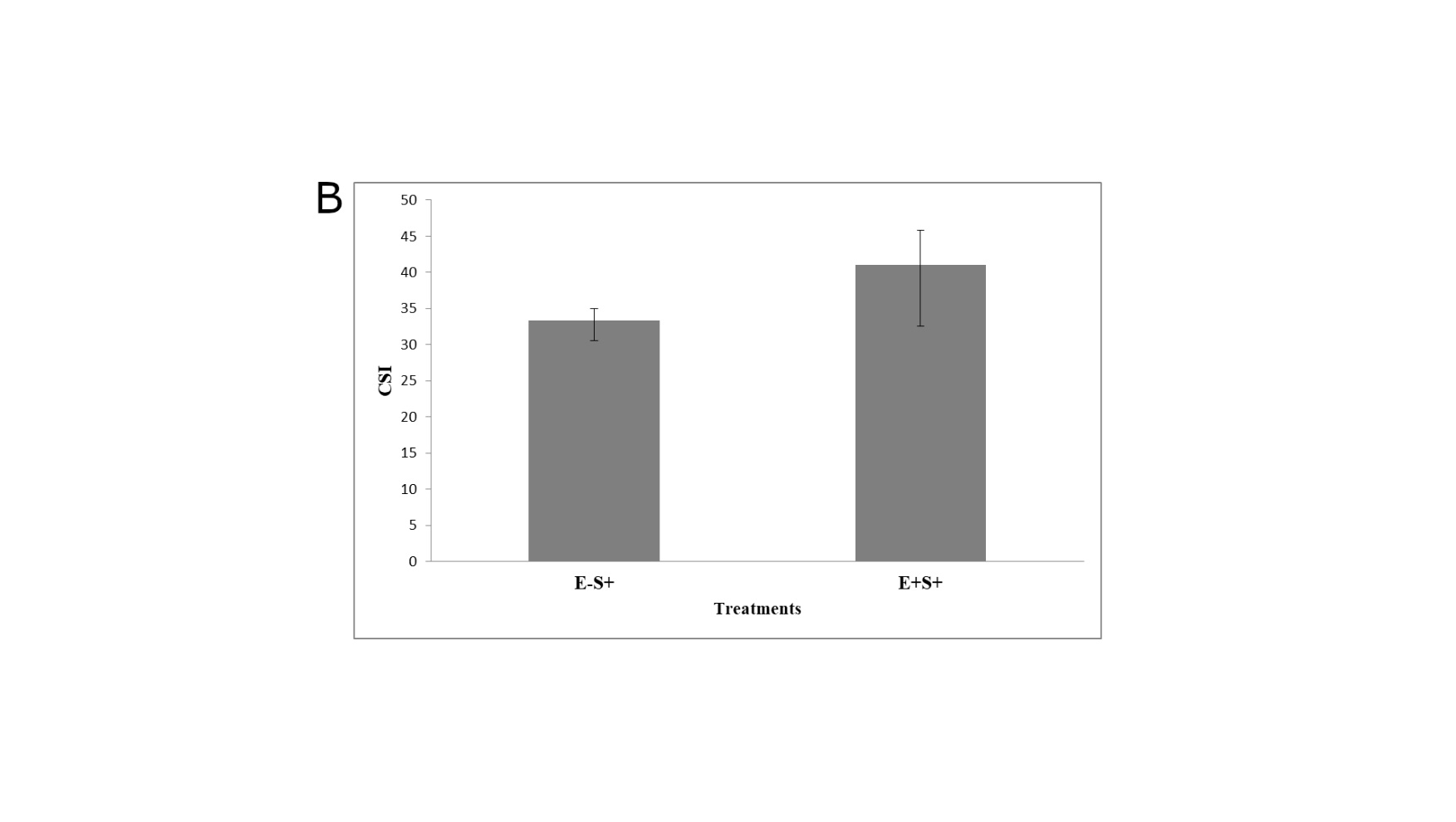

Supplement: Supplementary file 4 — Supplementary information4. [file 41598_2020_59998_MOESM4_ESM.docx]
