# Supplementary material for: An endophyte from salt-adapted Pokkali rice confers salt-tolerance to a salt-sensitive rice variety and targets a unique pattern of genes in its new host
Source: Sci Rep. 2020 Feb 24;10:3237. doi: 10.1038/s41598-020-59998-x (PMC7039991; doi:10.1038/s41598-020-59998-x)

**Figure S5** :RNA-Seq analysis between E-S+ and E+S+ treatments: A) Volcano plot representing DGEs – Blue dots (≥ 1 log2FC and ≤ -1 log2FC) and black dots (non significant log2FC)


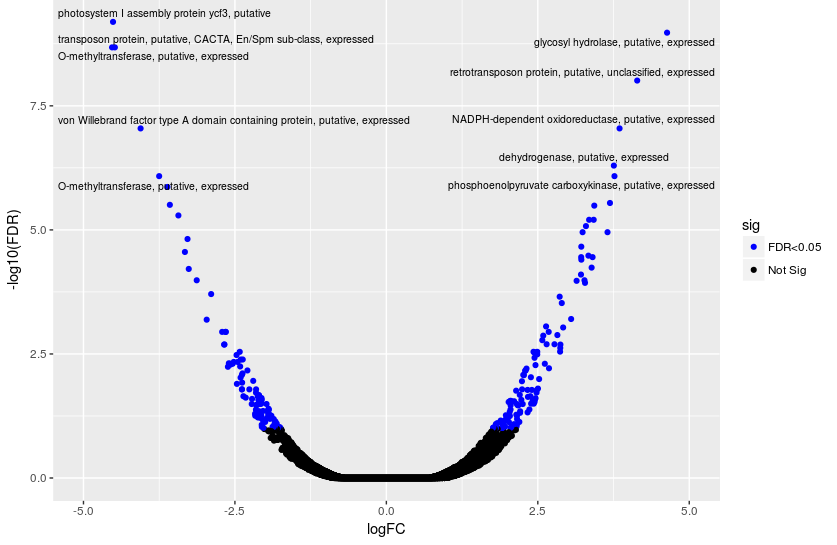

Supplement: Supplementary file 9 — Supplementary information9. [file 41598_2020_59998_MOESM9_ESM.docx]
