# Supplementary material for: An endophyte from salt-adapted Pokkali rice confers salt-tolerance to a salt-sensitive rice variety and targets a unique pattern of genes in its new host
Source: Sci Rep. 2020 Feb 24;10:3237. doi: 10.1038/s41598-020-59998-x (PMC7039991; doi:10.1038/s41598-020-59998-x)
Supplement: Supplementary file 10 — Supplementary information10. [file 41598_2020_59998_MOESM10_ESM.docx]

| **Raw reads** | | | **After filtering** | | **Reference assembly** | | | |
| --- | --- | --- | --- | --- | --- | --- | --- | --- |
| **Sample Name** | **Total raw reads** | **GC content (%)** | **% data**  **≥ Q30** | **HQ PE reads** | **Number of HQ reads aligned to reference genome** | **% aligned reads** | **Number of unaligned HQ reads** | **% Unaligned reads** |
| E+S+ | 92,624,402 | 45.1 | 91.2 | 84473454.62 | 75,181,354 | 81.50 | 17,069,214 | 18.5 |
| E-S+ | 104,343,026 | 46.8 | 91.3 | 95265182.74 | 73,269,907 | 73.25 | 26,758,275 | 26.75 |

**S10:** Summary statistics of transcriptome sequencing and assembly.
