# Supplementary material for: An endophyte from salt-adapted Pokkali rice confers salt-tolerance to a salt-sensitive rice variety and targets a unique pattern of genes in its new host
Source: Sci Rep. 2020 Feb 24;10:3237. doi: 10.1038/s41598-020-59998-x (PMC7039991; doi:10.1038/s41598-020-59998-x)
Supplement: Supplementary file 11 — Supplementary information11. [file 41598_2020_59998_MOESM11_ESM.docx]

| **Locus_ID** | **Present annotation MSU v 7.0** | **Protein of unknown function (PUFs) annotation** | **logFC** |
| --- | --- | --- | --- |
| LOC_Os03g10740.1 | expressed protein | Ubiquitin-like modifier-activating enzyme 1 | 3.270246 |
| LOC_Os10g21190.1 | expressed protein | Mevalonate pyrophosphate decarboxylase | 2.48542 |
| LOC_Os03g55680.1 | expressed protein | Myosin-2 heavy chain | 2.208537 |
| LOC_Os03g18250.1 | expressed protein | Phosphatidylinositol 3-kinase regulatory subunit beta | 2.195256 |
| LOC_Os11g29500.1 | expressed protein | Prelamin-A/C | 2.073324 |
| LOC_Os04g11120.1 | expressed protein | Ribonuclease 3 | 2.052984 |
| LOC_Os03g18270.1 | expressed protein | 361aa long hypothetical D-aminopeptidase | 1.961551 |
| LOC_Os11g02080.1 | expressed protein | Probable transmembrane ascorbate ferrireductase 2 | 1.94327 |
| LOC_Os10g29240.1 | expressed protein | Myc box-dependent-interacting protein 1 | 1.908015 |
| LOC_Os03g39655.1 | expressed protein | Secreted metalloprotease Mcp02 | 1.898262 |
| LOC_Os02g32580.1 | expressed protein | Histidinol dehydrogenase (1kae.A) | -2.1171 |
| LOC_Os12g32600.1 | hypothetical protein | Kemp eliminase KE59 R13 3/11H (3uzj.A) | -2.35988 |
| LOC_Os01g26039.1 | expressed protein | MGAT-like. Lysophospholipid acyltransferase (LPLAT) superfamily member | -2.38456 |
| LOC_Os03g51350.1 | expressed protein | CAP-Gly domain-containing linker protein 1 | -2.40344 |
| LOC_Os11g32890.1 | expressed protein | Talin-1(2jsw.A) | -2.64986 |
| LOC_Os12g08700.1 | expressed protein | Protein translocase subunit SecA 1(1nkt.A) | -3.13058 |
| LOC_Os12g33130.1 | expressed protein | Hyaluronidase, phage associated(2c3f.A) | -3.32546 |
| LOC_Os03g04080.1 | expressed protein | Hyaluronidase, phage associated(2c3f.A) | -3.43306 |
| LOC_Os03g42520.1 | expressed protein | Manganese peroxidase 1(3m5q.A) | -3.57406 |

**S11:** List of selected PUFs based on the logFC and its annotation after PUFA analysis.
