# Supplementary material for: An endophyte from salt-adapted Pokkali rice confers salt-tolerance to a salt-sensitive rice variety and targets a unique pattern of genes in its new host
Source: Sci Rep. 2020 Feb 24;10:3237. doi: 10.1038/s41598-020-59998-x (PMC7039991; doi:10.1038/s41598-020-59998-x)
Supplement: Supplementary file 12 — Supplementary information12. [file 41598_2020_59998_MOESM12_ESM.docx]

| **Sl.No** | **Gene name** | **Forward Sequence (5' - 3')** | **Reverse Sequence (5' - 3')** | **Tm (°C)** |
| --- | --- | --- | --- | --- |
| 1 | OsUBQ10 | TGGTCAGTAATCAGCCAGTTTGG | GCACCACAAATACTTGACGAACAG | 56 |
| 2 | OsIFR | CTCAACAGGCTCCTCTGAAATACA | CGGTGTGAAATTTGCAGGAA | 55 |
| 3 | OsWRKY | AACAGCGAGACGCAGTAAAAA | GAAACCAAAGGAGAGAAATCAAGA | 57 |
| 4 | OsCAM | AATGGTTTTATCTCTGCTGCTGAA | GATCTGCCCATCACCATCAAC | 57 |
| 5 | OsFBX | TTGGTAGCGAGATGAGGTGAA | GCACGGGACAAGCTGAGTAA | 56 |
| 6 | OsCa^2+^Sym | GATTCGGGTTGCCCTCTATG | CCCCACCATGCATCTTTAAA | 59 |
| 7 | OsBHLH | TGGAATGCAGCTCCTTTGAA | GTCGTAGCCCTCACTACTGATGAG | 56 |
| 8 | OsNa^+^Sym | TGTTTGTGCCGTTGATTCTG | CTTGCTCATAGGCCCCATCT | 55 |
| 9 | OsSAM | GTTACCCTTCCCTAGTGGTGCATT | CTCCTGGACGAAGGATCCTATTTATC | 57 |
| 10 | OsORD | CCCACTCCAGTCGTTGCTTT | AGATGCCATTCGTTTGATGAGA | 58 |
| 11 | OsNHX | GAGGAGAGCCGATGGATGAA | GCGGGAGCAGGTAAATGAAG | 59 |
| 12 | OsRING | CCTGGAATTGGTGCTGAACA | ACCCCCAGGCAGTATACAGA | 58 |
| 13 | OsHKT | GCTCTGGACTGCGGTTGGTATT | GCTTCGCAAGTGGCATAAGAGA | 60 |
| 14 | OsPOD | CGGACGCCCAACACCTT | TGGTGGTTGCGTTGAAGAAG | 59 |
| 15 | OsRLK2 | CCGGCTCCTACGGCTACAT | GGGTACTTGCCCATGAGCAT | 59 |
| 16 | OsbZIP | CAATGGAATGAGTGTGCGAGTTT | CTCCGGTTCTGATGCCTTTG | 60 |
| 17 | ITS1/ITS4 | tccgtaggtgaacctgcgg | tcctccgcttattgatatgc | 55 |

**Table S12:** Primers used in the study: (1) Endogenous gene, (2-10) qPCR analysis for relative quantification; (11-16) primers which did not have slope of – 3.3 for relative quantification and (17) Molecular characterization of the OTUs
